# Shedding and stability of CWD prion seeding activity in cervid feces

**DOI:** 10.1371/journal.pone.0227094

**Published:** 2020-03-03

**Authors:** Joanne M. Tennant, Manci Li, Davin M. Henderson, Margaret L. Tyer, Nathaniel D. Denkers, Nicholas J. Haley, Candace K. Mathiason, Edward A. Hoover

**Affiliations:** 1 Prion Research Center, College of Veterinary Medicine and Biomedical Sciences, Colorado State University, Fort Collins, CO, United States of America; 2 Midwestern University, College of Graduate Studies, Glendale, AZ, United States of America; National Institute of Allergy and Infectious Diseases, UNITED STATES

## Abstract

CWD is an emergent prion disease that now affects cervid species on three continents. CWD is efficiently spread in wild and captive populations, likely through both direct animal contact and environmental contamination. Here, by longitudinally assaying in feces of CWD-exposed white-tailed deer by RT-QuIC, we demonstrate fecal shedding of prion seeding activity months before onset of clinical symptoms and continuing throughout the disease course. We also examine the impact of simulated environmental conditions such as repeated freeze-thaw cycles and desiccation on fecal prion seeding activity. We found that while multiple (n = 7) freeze-thaw cycles substantially decreased fecal seeding activity, desiccation had little to no effect on seeding activity. Finally, we examined whether RT-QuIC testing of landscape fecal deposits could distinguish two premises with substantial known CWD prevalence from one in which no CWD-infected animals had been detected. In the above pilot study, this distinction was possible. We conclude that fecal shedding of CWD prions occurs over much of the disease course, that environmental factors influence prion seeding activity, and that it is feasible to detect fecal prion contamination using RT-QuIC.

## Introduction

Chronic wasting disease (CWD) is an emergent prion disease, or transmissible spongiform encephalopathy (TSE), that affects free-ranging and captive cervid populations, including elk, deer, reindeer, and moose. CWD is primarily found in North America, and since its discovery in the late 1960s it is now identified in 26 states in the United States, two Canadian provinces, South Korea, Norway, Finland and Sweden [1, 2](www.nwhc.usgs.gov). As with other prion diseases, CWD is caused by a misfolded, protease-resistant pathogenic form (PrP^Sc^) of the normal cellular protein (PrP^C^) [3–6].

Transmission of CWD is efficient, yet somewhat enigmatic. Direct and indirect (environmental) horizontal transmission appear to be the principal modes of CWD spread, but pre- and peri-natal transmission also are likely [7, 8]. Shedding of CWD prions or prion seeding activity has been demonstrated in saliva, urine, and feces of asymptomatic and symptomatic deer and elk [8–17]. Depopulation/repopulation studies provided the first support that habitats of cervids contaminated by CWD prions facilitate CWD transmission [18, 19]. Yet, paradoxically, infection has been difficult to produce in naïve deer by experimental oral inoculation of urine or feces from CWD-infected donors [20].

Studies demonstrating CWD prion shedding in secretions, excretions, and the environment have been challenging due to the low concentrations of prions in these materials, below that demonstrable by western blotting, enzyme-linked immunosorbent assay (ELISA), or even bioassay [15, 20]. Development of sensitive PrP^Sc^ amplification methods, including the serial protein misfolding cyclic amplification (sPMCA) and real-time quaking-induced conversion (RT-QuIC), has permitted the detection of prion seeding activity with sensitivity beyond that demonstrable in even bioassays [21–25]. However, the complex biologic milieu in excreta contains assay inhibitors and/or non-specific activators that can interfere with in vitro amplification assays [26, 27]. Nevertheless, through modifications of assay conditions, assays such as RT-QuIC can deliver sufficient sensitivity and specificity in problematic biologic samples [12, 26, 28, 29].

Here we have examined longitudinal prion shedding in feces of white-tailed deer exposed orally to low infectious doses of CWD. Since environmental conditions (e.g. drying, freezing) have been shown to have variable effects on prion biologic activity [30, 31], we examined the effects of these influences on retention of CWD prion seeding activity in cervid feces. Finally, we extended this work to the natural landscape in a pilot study examining blinded fecal samples from premises containing CWD positive vs. CWD negative animals. Our findings demonstrate that fecal CWD prion seeding activity is shed throughout the disease course, this activity can be affected by simulated environmental conditions, and that it is feasible to detect landscape fecal prion contamination using RT-QuIC.

## Results

### Detection of fecal prion seeding activity in deer with low-dose CWD infection

**Fig 1:**
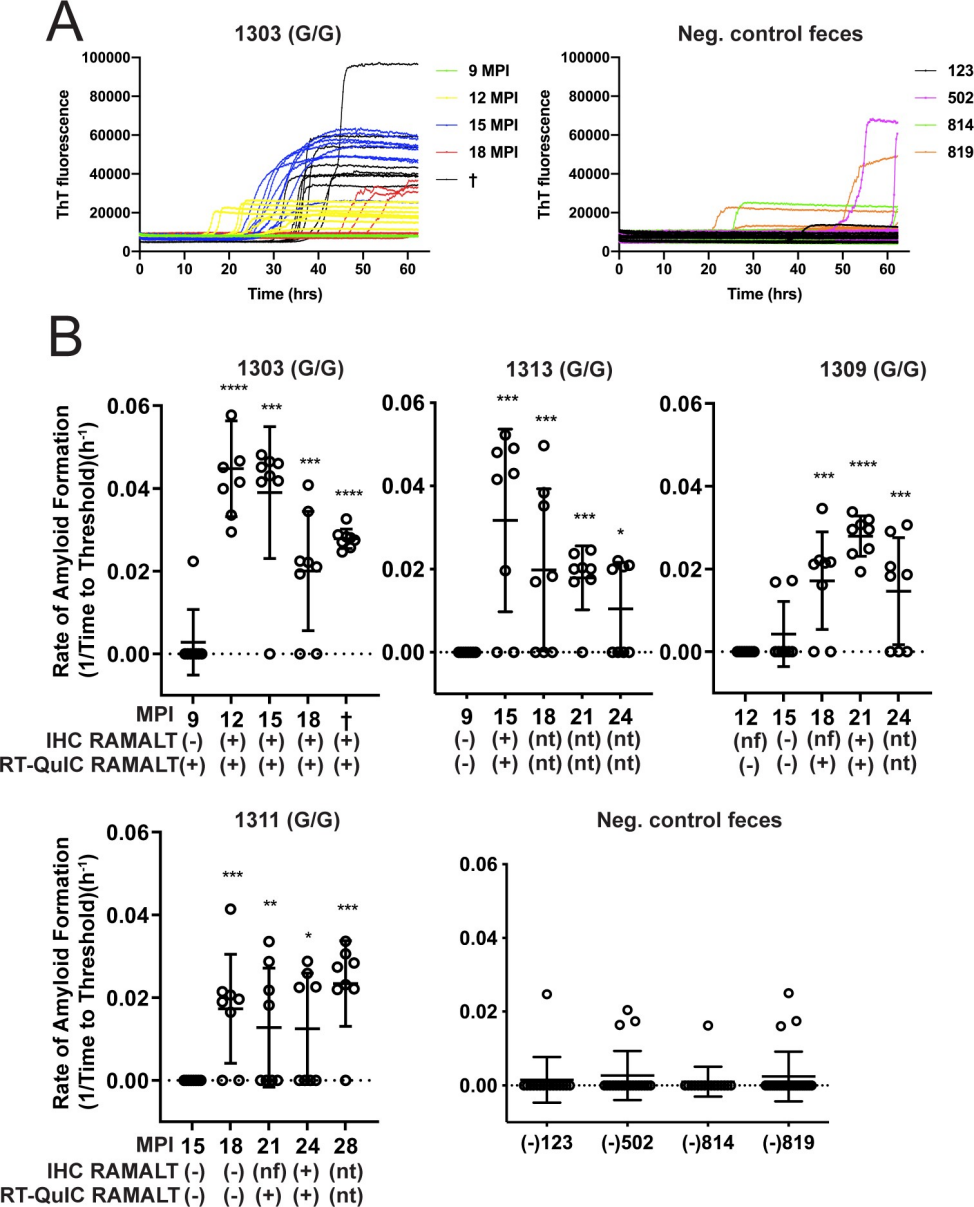
Longitudinal CWD prion fecal shedding in 96 GG deer. **(**A) Representative RT-QuIC data curves from CWD positive deer, 1303, at multiple sampling timepoints and negative controls. Data from these graphs is converted to reaction rates by 1/time to cross the threshold (5 SD above the mean background) and shown below. (B) Five collection time points encompassing shedding of fecal prions are displayed for deer 1303, 1313, 1309, and 1311—a representative cohort of 96GG deer in the study. Y-axis displays amyloid formation rate for CWD seeding activity at each sampling time point displayed on the x-axis as months post inoculation (MPI). Below MPI, IHC and RT-QuIC data are displayed for RAMALT biopsies taken at the same collection. IHC or RT-QuIC testing nt: signifies that no sample was taken for IHC or RT-QuIC due to positive results on a prior test. Nf: signifies that no follicles were present in the biopsy following microscopic examination and therefore no IHC interpretation could be conducted. Negative control fecal samples were also tested from deer that were CWD negative (deer # 123, 502, 814, and 819). Data shown is the amyloid formation rate of 8 replicates with mean ± SEM; * p<0.05, ** p<0.01, *** p<0.001, **** p<0.0001.

**Fig 2:**
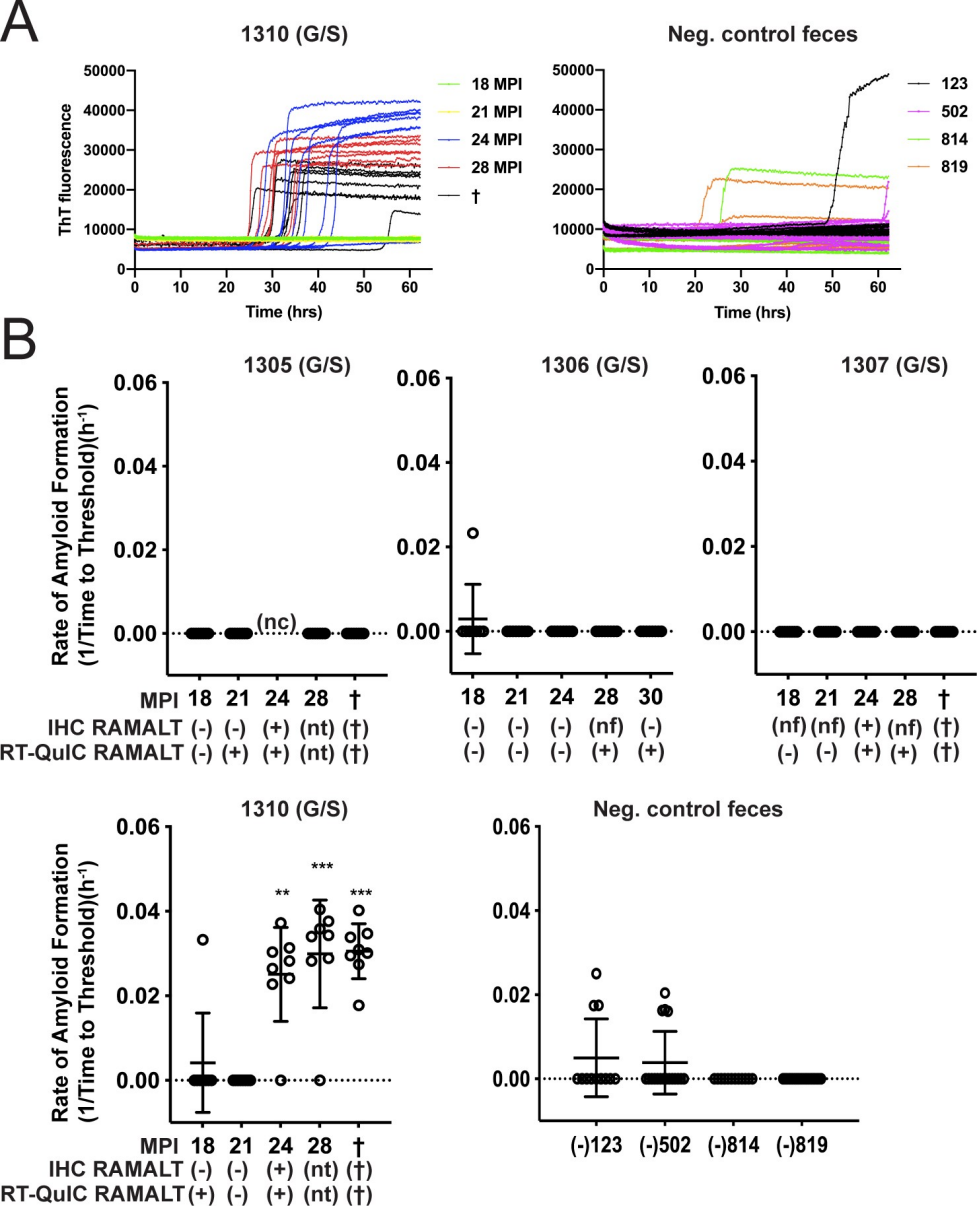
Longitudinal CWD prion fecal shedding in 96 GS deer. **(**A) Representative RT-QuIC data curves from CWD positive deer, 1310, at multiple sampling timepoints and negative controls. Data from these graphs is expressed as reaction rates (1/time to cross threshold) as described previously and shown below. (B) Five collection time points encompassing fecal prion shedding are displayed for deer 1305, 1306, 1307, and 1310. Y-axis displays amyloid formation rate for CWD seeding activity at each sampling point displayed on the x-axis as months post inoculation (MPI). Below MPI, IHC and RT-QuIC data are displayed for RAMALT biopsies taken at the same collection. For IHC or RT-QuIC testing in which nt is entered, no sample was taken due to previous positive IHC results. For IHC, nf signifies that no follicles were present in the biopsy sample. For RT-QuIC feces, nc denotes that no sample was collected at the timepoint. Negative control fecal samples were also included, which were collected from CWD negative deer (deer # 123, 502, 814, and 819). Data shown is the amyloid formation rate of 8 replicates with mean ± SEM; * p<0.05, ** p<0.01, *** p<0.001, **** p<0.0001.

Previous studies have demonstrated that CWD seeding activity and infectivity can be detected in feces, inferring its contribution to environmental CWD contamination [8, 12, 15, 32]. With the goal of acquiring an overall profile of CWD shedding, we longitudinally monitored prion seeding activity in feces collected from white-tailed deer orally infected with low doses (300ng to 1mg of CWD positive brain equivalents) of CWD prions in brain or saliva. Fecal prion seeding activity was detected by RT-QuIC from 12 to 18 months post CWD exposure in 5 of 6 codon 96GG deer and after 24 months in 1 of 4 96GS deer (Figs 1 and 2). In the 4 96GG deer shown, the first instance of IHC positivity in RAMALT tissue biopsy correlated with the first detection of fecal positivity by RT-QuIC (Fig 1). In all animals, seeding activity, once detected, remained detectable throughout preclinical and clinical disease course (Figs 1 and 2).

### CWD fecal prion seeding activity is not significantly affected by desiccation

**Fig 3:**
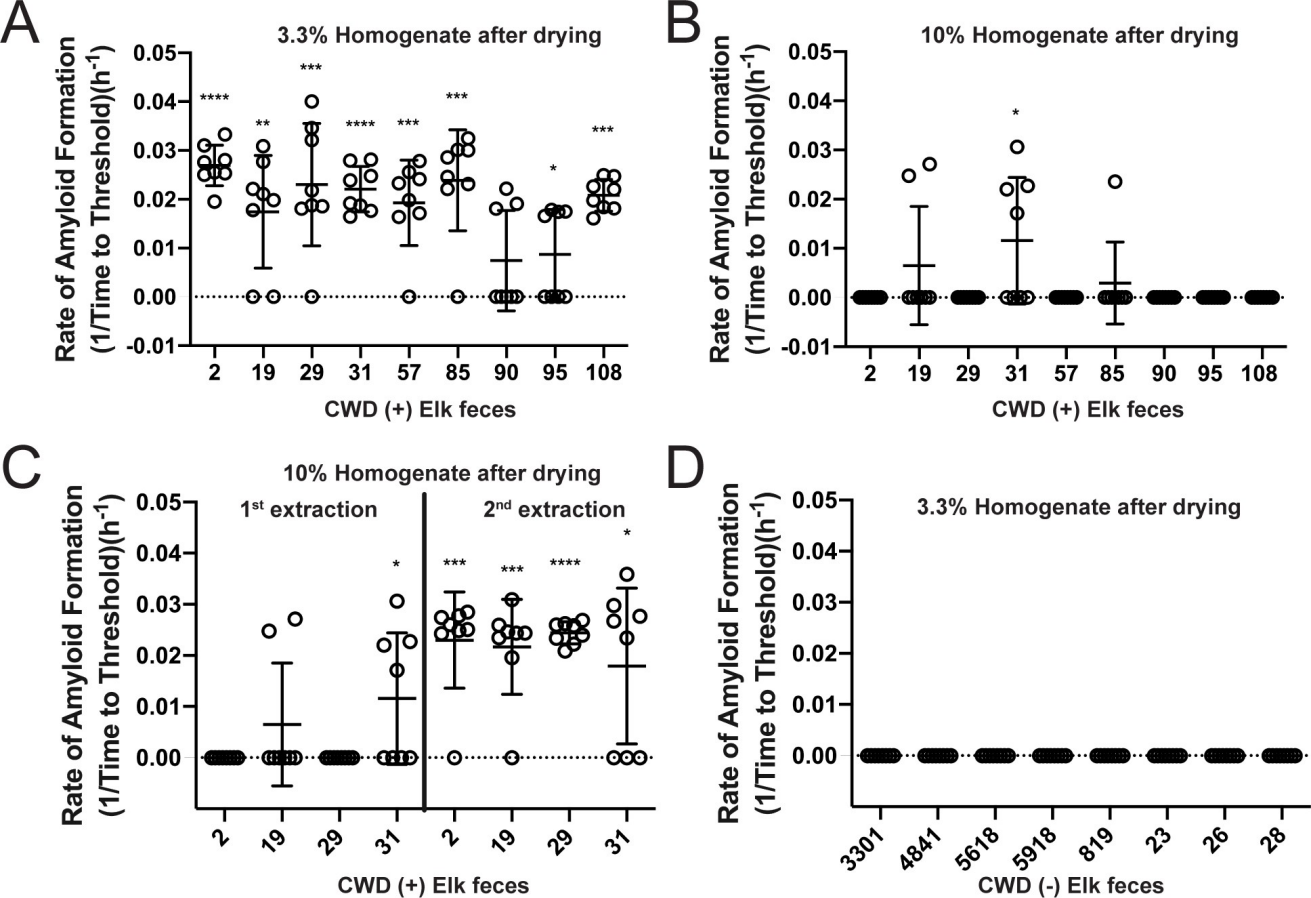
Desiccation of CWD positive fecal samples does not affect seeding activity. CWD positive elk fecal samples’ rate of amyloid formation (y-axis) were analyzed to ascertain the seeding activity after drying (A-C). (A) Fecal homogenates prepared as a 3.3% homogenate based on dry weight (approximating a 10% homogenate based on wet weight) retained seeding activity after desiccation. (B) When fecal samples were prepared as 10% homogenates based on dry weight, inhibition of positive seeding was observed. (C) If fecal samples were extracted a second time as a 10% homogenate, there was reduced inhibition of seeding activity. (D) Fecal samples from CWD positive elk were not affected by desiccation or homogenization at a dilute concentration (3.3% homogenate dry weight or approximating a 10% homogenate wet weight). Data shown is the amyloid formation rate of 8 replicates with mean ± SEM; * p<0.05, ** p<0.01, *** p<0.001, **** p<0.0001.

Because fecal deposit on the landscape are subjected to desiccation, UV radiation, and freeze/thaw cycles, we sought to determine the potential effects of these conditions on prion seeding activity. Feces from rocky mountain elk naturally infected with CWD were subjected to desiccation for 72 hours, during which they lost an average of 66% of their wet weight. After drying, the fecal samples were homogenized in phosphate buffered saline (PBS) based on either their wet or dry weights. We found that CWD seeding activity was retained when desiccated fecal samples were homogenized based on their wet weight (yielding a 3.3% homogenate, or 33.3 mg of dry feces per 1 ml of PBS) (Fig 3A). However, when samples were homogenized based on their dried weight (equaling a 10% homogenate, 100 mg of dry feces per 1 ml of PBS), we observed a decrease in RT-QuIC prion seeding activity (Fig 3B). However, a second dilution and NaPTA precipitation of the 10% homogenate recovered prion seeding activity in samples previously seen to have little seeding activity (Fig 3C). Thus, the higher fecal concentration in 10% homogenates resulted in inhibition of the RT-QuIC assay as seen by a loss of seeding activity. Further dilution did not affect false-positivity in the RT-QuIC assay as 3.3% fecal homogenates (33.3 mg of dry feces per 1 ml PBS) from CWD negative deer did not exhibit seeding activity in RT-QuIC assays (Fig 3D).

### CWD fecal prion seeding activity is diminished by multiple freeze/thaw cycles

**Fig 4:**
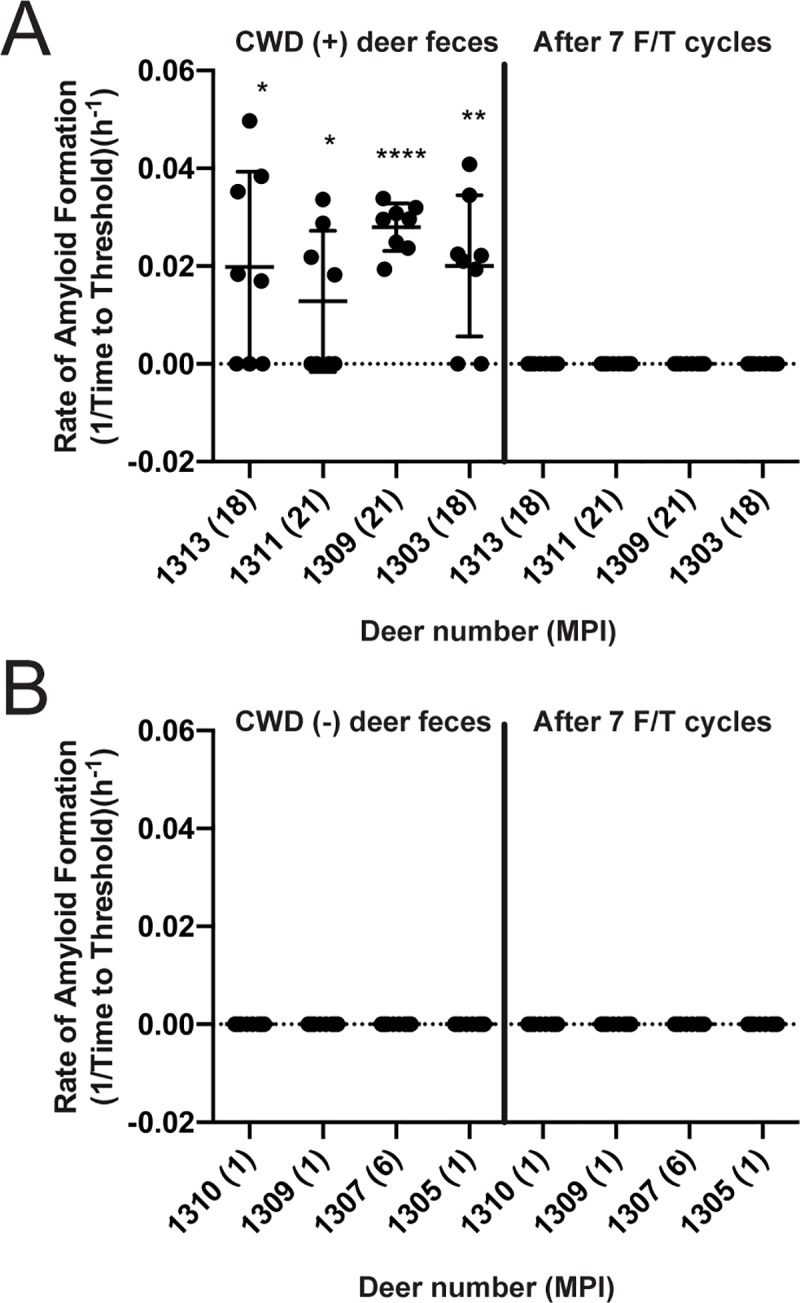
CWD seeding activity is decreased by seven rounds of freezing and thawing of deer fecal pellets. Deer fecal pellets were tested prior to freezing and thawing and then after 7 cycles of freeze/thaw. Graphs show amyloid formation rate from feces of deer at certain collection timepoints post infection. (A) Fecal samples from CWD positive deer initially tested positive but seeding activity was lost after 7 freeze/thaw cycles. (B) Fecal samples from CWD negative deer were affected by freeze/thaw as all negative for seeding activity initially and remained negative after 7 freeze/thaw cycles. Data shown are the amyloid formation rate of 8 replicates with mean ± SEM; * p<0.05, ** p<0.01, *** p<0.001, **** p<0.0001.

**Fig 5:**
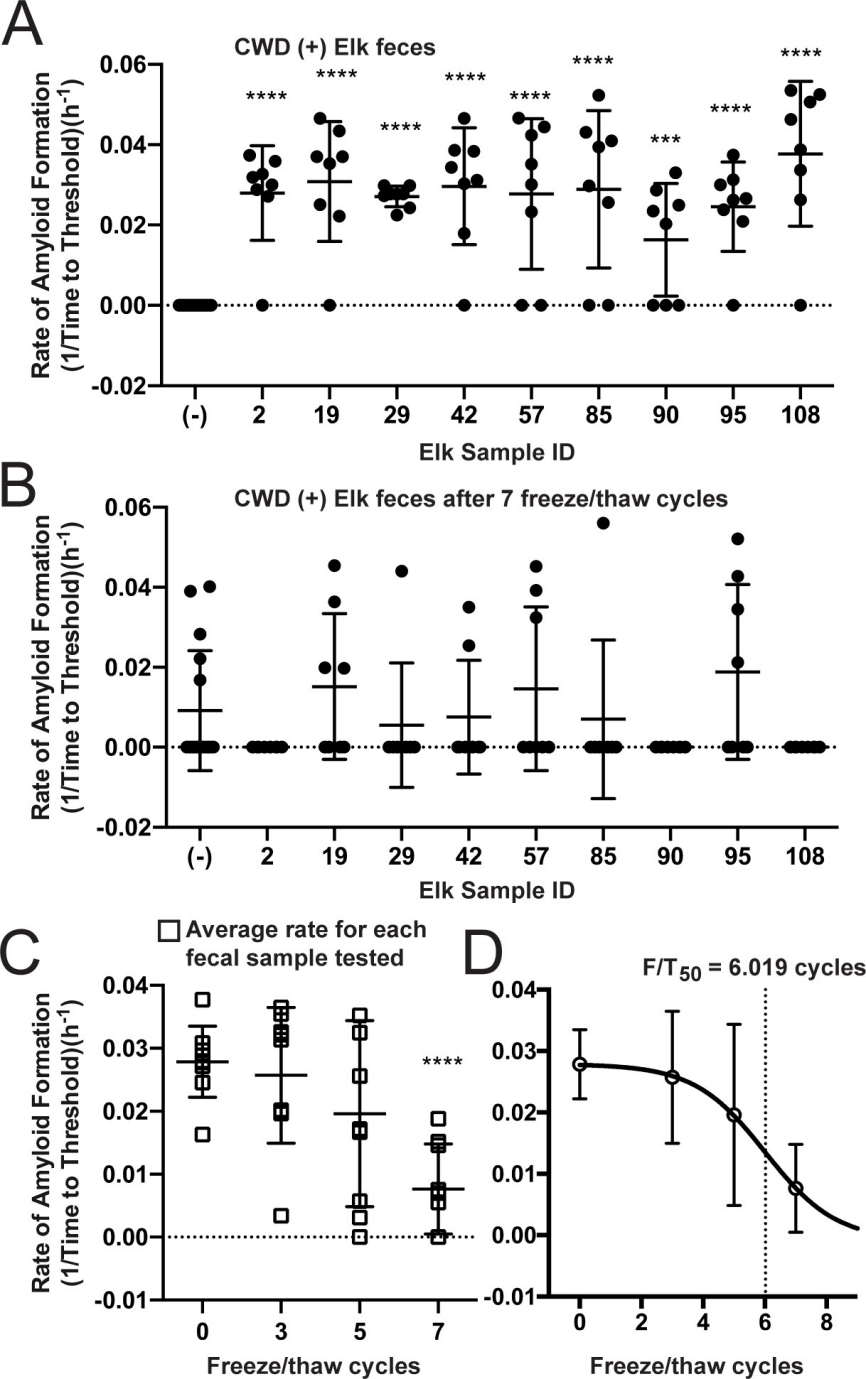
CWD seeding activity is decreased in elk fecal pellets through multiple rounds of freeze/thaw cycles. (A-B) All 9 elk fecal pellets positive for CWD seeding activity were reduced to background levels of seeding after 7 rounds of freeze/thaw and an increase in non-specific reaction was seen in CWD negative samples (-). Data shown (A-B) is the amyloid formation rate of 8 replicates with mean ± SEM. (C) Mean seeding activity of each elk pellet at select freeze/thaw cycles was plotted and a reduction in mean seeding activity of samples was observed as the number of freeze/thaw cycles increases. (D) Mean seeding activity from all samples after selected freeze/thaw cycles (C) were averaged and fit with Boltzmann sigmoidal cure to determine a point where 50% of the seeding activity is lost. F/T_50_ = 6.019. * p<0.05, ** p<0.01, *** p<0.001, **** p<0.0001.

To simulate the potential effect of freeze/thaw cycles in the natural environment, we subjected fecal pellets from CWD positive and negative cervids to serial cycles of freezing at -20°C and thawing at room temperature. We used fecal samples collected from both indoor-housed white-tailed deer and free-ranging elk. CWD seeding activity feces from CWD-infected deer was serially diminished by successive freeze/thaw cycles and was extinguished after 7 freeze/thaw rounds (Fig 4A). Parallel freeze/thaw cycles on feces from CWD negative deer had no effect (Fig 4B). We observed a similar pattern of decrease and extinction trajectory in CWD seeding activity in elk feces after 7 freeze/thaw cycles (Fig 5A and 5B) with each freeze/thaw cycle decreasing the mean seeding activity (Fig 5C). By fitting the averaged mean seeding activities for each of 9 elk fecal samples to a Boltzmann sigmoidal curve, we determined the point at which 50% of the seeding activity was lost (F/T_50_) was 6.019 cycles. We also observed an increase in non-specific/false positive seeding results in CWD positive elk after 7 freeze/thaw cycles (Fig 5B).

### Detection of CWD prion seeding activity in feces from CWD endemic and non-endemic landscapes

**Fig 6:**
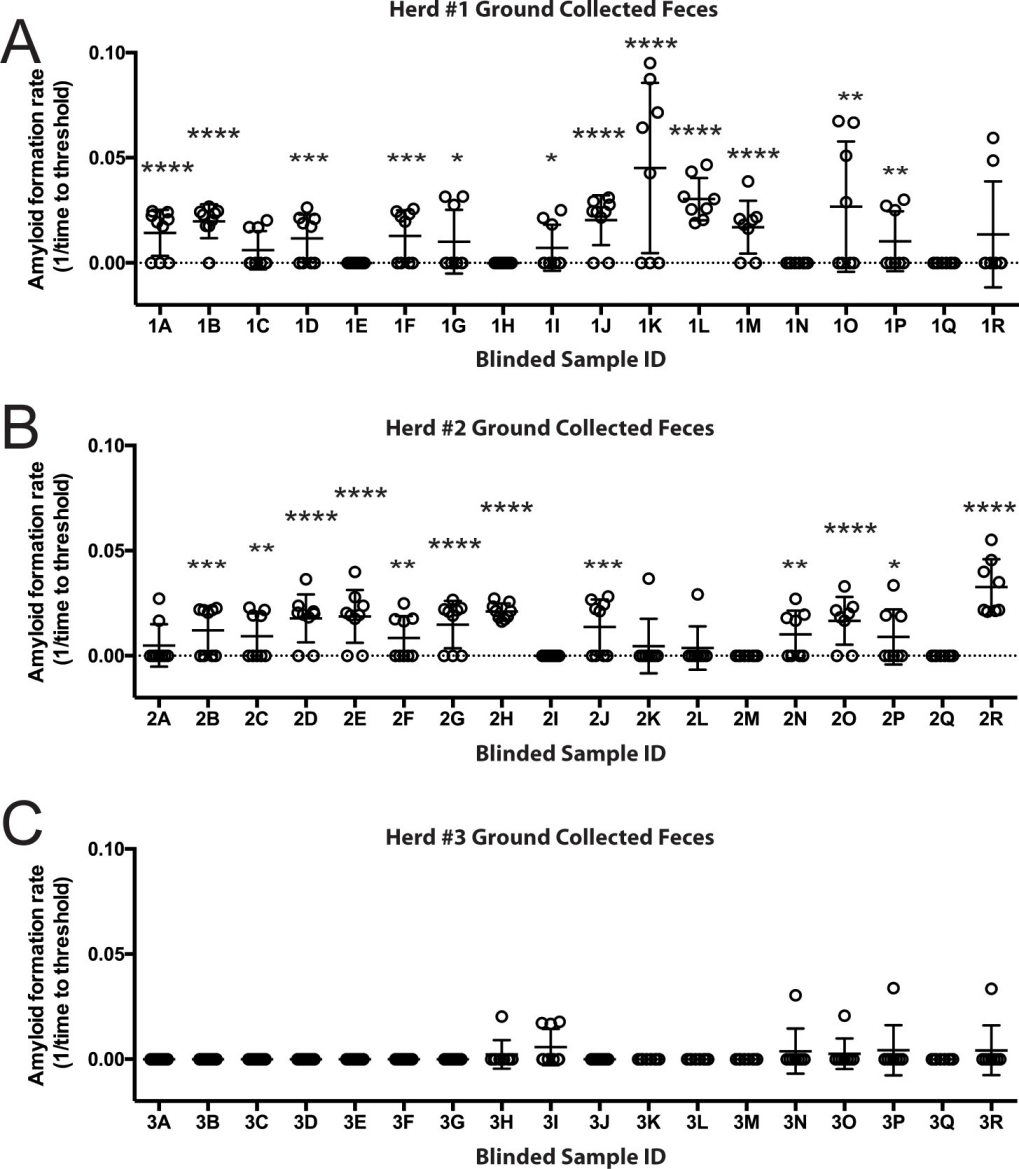
Blinded evaluation of environmental fecal samples collected from CWD positive and negative herds. Freshly deposited feces were collected from the ground at three different deer ranches. (A) Seeding activity of feces collected Ranch #1, with ~50% CWD prevalence. Twelve of 18 samples demonstrated seeding activity significantly higher than that of negative controls; * p<0.05, ** p<0.01, *** p<0.001, **** p<0.0001. (B) Ground-collected feces from Ranch #2 demonstrated significant seeding activity in 12 of 18 samples tested. Ranch #2 had CWD prevalence of about 30% from the pens where feces were primarily collected. (C) Ranch #3, a CWD negative herd over 100 miles from the nearest reported case of CWD in wild deer, showed no significant seeding activity in ground collected fecal samples as compared to negative controls.

To test whether RT-QuIC analysis of feces could provide insight into the CWD status of a deer population, we blindly analyzed samples collected from the landscape of three separate white-tailed deer populations, two with known prevalence of CWD and a third wherein no CWD positive deer had been encountered. While the CWD status of specific source deer leaving the fecal pellets was unknown, each premises had active CWD surveillance conducted via postmortem testing. Eighteen randomly collected fecal samples, each from separate deposits, were evaluated from each of the three study sites. Analysis of samples from ranch #1, with an estimated postmortem CWD prevalence of 50%, identified 12 of 18 samples (66.6%) as positive for CWD seeding activity (Fig 6A). RT-QuIC analysis of fecal samples from ranch #2 also detected 12 of 18 (66.6%) positives. Pens from which fecal samples were collected from ranch #2 had an approximate CWD prevalence of 30% (Fig 6B). Ranch #3 was located in a CWD negative area over 100 miles from any known CWD occurrence and had no history of CWD on site. None of the 18 fecal samples tested from this site tested positive (Fig 6C). These initial results suggest that RT-QuIC analysis of ground-collected, fresh fecal samples may be useful in identifying landscapes that contain a substantial number of CWD positive animals.

## Discussion

This study demonstrates that deer inoculated with low but infectious doses of CWD, potentially more closely emulating natural exposure, shed CWD prions in feces over much of the disease course. The onset of fecal shedding approximated the time of first IHC positivity in RAMALT biopsies (12–24 months post-exposure, depending on codon 96 genotype) (Figs 1 and 2). While prion infectivity [15] or seeding activity by PMCA [33] has been demonstrated previously in feces from CWD-infected deer as well as scrapie-infected hamsters [14, 34], to our knowledge, this is the first longitudinal examination of fecal shedding in relation to the onset of detectable systemic infection.

We observed decreased shedding in deer with the 96GS polymorphism compared with 96GG deer. In 5 of 6 (83.3%) 96GG deer, shedding of fecal CWD seeding activity was concurrent with first IHC positivity in RAMALT biopsies; this contrasted with 1 of 4 (25%) 96GS deer. Although limited by small numbers, these results are consistent with those of Plummer et al. [33] who found that susceptible 96GG deer were shedding CWD prions in feces more than resistant genotypes (96GS and 96SS) with slower disease kinetics [35].

Our results exploring the effects of simulated environmental conditions on prion activity correlate well those of the Bartz [30, 31] laboratory using brain-derived CWD and TME prions and different assay systems—PMCA, western blotting, and infectivity. Interestingly, the above studies [31] also demonstrated that prion binding to soil constituents allowed prions to resist the detrimental effects of environmental conditions on seeding activity. Given the low levels of prion shedding in feces, detailed below, binding to soil constituents may be important in environmental persistence and transmission of CWD.

We have previously reported that relative to prion seeding levels in target tissues, prion seeding concentrations in feces and other secretions and excretions are low—at least 5 log_10_ [12, 13]. The low detectable level of prions in excreta samples coupled with higher frequency of non-specific rPrP aggregation, poses challenges in both sensitivity and specificity when assaying feces [12, 36, 37]. However, by using NaPTA to concentrate fecal prions and reduce non-specific reactivity, we were able to distinguish differences in positive and negative deer with sufficient specificity to achieve statistical significance. While we were able to detect seeding activity throughout clinical disease course, we did not see an increase in activity rates in terminal stages as reported in a previous study from our laboratory [12].

Despite the findings of amplifiable prions in feces, attempts to transmit infection by oral exposure of naive deer to feces from CWD-positive donors have failed to produce overtly detectable infection [13]. Nevertheless, given the wealth of evidence that CWD (and scrapie) can be transmitted by exposure to contaminated environments [38, 39], further studies are needed to better understand the enigmas and the mechanisms of CWD transmission in nature.

In hope of gaining insight into whether the in vitro modeling studies described here have application to natural settings, we sampled feces from three landscapes and demonstrated that RT-QuIC analysis could discriminate premises with high levels of CWD from a CWD negative location. Ground-collected feces from the two sites harboring CWD-positive cervids demonstrated prion seeding in RT-QuIC whereas the ranch site with no known CWD-positive animals did not. Other studies have demonstrated detection of prion seeding activity, or in one instance, infectivity [15], in feces from naturally or experimentally infected cervids [32, 33, 37, 40]. However, none have used ground-collected feces as a potential indicator of CWD positive status of a premises or cervid population. Taken together, these findings suggest that RT-QuIC, and potentially other amplification assays, could be used as a CWD surveillance tool.

In conclusion, we demonstrate that CWD prion seeding activity can be identified in feces freshly collected directly from cervids and in recently deposited feces collected from landscape sites known to harbor CWD-infected deer. We also show that repeated freezing and thawing of feces may impact detection of shed prions in feces. Further studies are needed to better understand CWD prion stability in the environment.

## Materials and methods

### Deer inoculation and sample collection

The deer studies in the manuscript were approved by the Institutional Animal Care and Use Committee at Colorado State University. The Warnell School of Forestry and Natural Resources, University of Georgia supplied white-tailed deer (*Odocoileus virginianus*) from a CWD negative region for use in the captive deer studies. The deer were transferred to the indoor CWD research facility at Colorado State University. Deer were orally inoculated with 300ng or 1mg of CWD positive brain or 30 ml of saliva, as published [41]. Feces were collected with fresh gloves from the rectum of anesthetized deer and stored at -80°C until testing. Feces from farmed elk (*Cervus elaphus elaphus*) with an approximate herd CWD prevalence of 35% were collected with fresh gloves from the rectum during yearly elk inventory [42]. A modern, conventional animal handling system was used and the elk were minimally restrained during sampling. These elk ranged on approximately 3000 acres of fenced habitat that resembles free-range elk habitat in the Rocky Mountains. Finally, fresh white-tailed deer fecal pellets were collected from the ground using fresh gloves at three separate locations, two where CWD was highly endemic and a third over 100 miles from the nearest reported case of CWD in wild deer. The ranch #1 herd is made up of approximately 80 deer maintained on approximately 360 acres of fenced land comprised of 270 acres of forested land and 90 acres of fields. The second herd (ranch #2) is located on 480 acres of fenced wooded land with eight small 2–3 acre pens inside the larger fenced acreage. There are ~250 animals on the property and ~50 animals in the pens; fecal samples were collected almost entirely from the penned area. The ranch #3 herd is a breeding herd of ~120 deer on 25 acres, and to date CWD has not been reported in any members of the herd. Negative control elk feces were donated by Theodore Roosevelt National Park from culled elk in the park. Feces were collected from the rectum shortly after death in the same manner as terminal deer feces collection.

### Sample preparation

Samples were stored at -80°C after collection and until testing. Whole or portions of fecal pellets were weighed and Phosphate Buffered Saline (PBS) was added to make a 10% homogenate for rectal collected samples. For desiccation studies, feces were weighed prior to drying for 72 hours in a hood with both the blower and UV lamp on. They were then homogenized to 10% w/v of the dried weight, or 3.3% w/v of the dried weight, which corresponded to an average of 10% w/v of the rectal collected weight. Frozen and thawed feces were subjected to 24 hours of freezing at -20°C prior to 24 hours at room temperature on a benchtop. All fecal pellets were manually disrupted and vortexed to make a homogenized solution that was centrifuged at 3,000 rpm for 15 minutes. The supernatant was collected and 500 μl was centrifuged at 14,000 rpm for 30 minutes with the supernatant of the spin discarded. The pellet was resuspended in 100 μl of PBS and incubated with 7 μl of NaPTA solution (0.5 g sodium phosphotungstic acid, 0.43 g MgCl_2_-6-hydrate) at 37°C and continuously shaken at 1,400 rpm for 1 hr. Samples were then centrifuged for 30 minutes at 14,000 rpm. The supernatant was discarded and the pellet was resuspended in 10 μl 0.05% SDS in PBS and 2 μl of the suspension was placed in each reaction well. Four reactions were run from each preparation and the experiment was repeated so that each sample had 8 replicates.

### RT-QuIC substrate preparation

RT-QuIC testing was performed with recombinant Syrian hamster PrP^C^ (SH-rPrP) truncated to residues 90–231, expressed and purified as previously written [11]. Briefly, SHrPrP was expressed in BL21 Rosetta *Escherichia coli* (Novagen) cultured in lysogeny broth (LB) at 37°C in the presence of selection antibiotics and auto induced overnight. Cells were harvested and lysed with BugBuster and Lysonase (EMD-Milipore). The resultant inclusion bodies were solubilized in 8.0 M guanidine hydrochloride (GdnHCl) with 100 mM NaPO_4_, mixed at room temperature and bound to Superflow Ni resin (GE). SH-rPrP (90–231) was refolded with an increasing linear gradient of 100 mM NaPO_4_, 10 mM Tris pH8.0 and a subsequent decrease in 6 M GdnHCl, 100 mM NaPO_4_, 10 mM Tris pH8.0. Finally, the protein was eluted with a linear gradient of 100 mM NaPO_4_, 10 mM Tris, and 0.5 M Imidazole pH 5.5. The eluted protein was dialyzed overnight in two changes of 20 mM NaPO_4_ pH 5.5. As previously described, the final protein concentration was determined by spectrophotometer at A280 and stored at 4°C for no longer than one month.

### RT-QuIC protocol

RT-QuIC experiments were carried out as previously described [11, 35]. Briefly each well in 96-well, optical bottom plates (Greiner) contained 0.1 mg/ml SH-rPrP (90–231) and RT-QuIC reaction buffer (20 mM NaPO_4_, 320 mM NaCl, 1.0 mM ethylenediaminetetraacetic acid, and 1 mM thioflavin T). Reaction plates with SH-rPrP, RT-QuIC reaction mix, and sample were loaded into a BMG Polarstar ^TM^ fluorometer/plate reader and the experiment performed at 37°C. Plates were subjected to cycles of shaking at 700 rpm for one minute and followed by one minute of rest. After 15 minutes, fluorescent readings with an excitation of 450 nm and emission of 480 nm were taken using 20 flashes and an orbital average of four. Each experiment consisted of 250 cycles of fluorescent reading and an experimental well was deemed positive if the fluorescence in the well surpassed 5 standard deviations of the mean baseline of all 96 wells. The time it took a sample to become positive was determined by the time-to-threshold calculator in the BMG Mars software and was used to determine rate of amyloid formation. As previously described, rate of amyloid formation is equivalent to one divided by the time-to-threshold and the log-linear regressions were plotted in GraphPad Prism [43]. A Mann-Whitney T-Test was used to determine if samples were positive compared to the negative plate controls.
